# General Practitioners' Perceptions of Patient Involvement—An Interview Study

**DOI:** 10.1111/jep.70077

**Published:** 2025-04-06

**Authors:** Birgitte Nørgaard, Elisa Simonsen, Nanna Aarup Skotte, Michael Marcussen

**Affiliations:** ^1^ Department of Public Health University of Southern Denmark Odense Denmark

**Keywords:** experience, healthcare, patient‐centered care, person‐centered medicine, primary care, qualitative methods

## Abstract

**Rationale and Aim:**

General practitioners (GPs) play an increasingly important role in the healthcare system, wherein patient involvement is a key element in delivering individualized and tailored treatment. This study aimed to explore GPs' perceptions of user involvement and their considered challenges and opportunities regarding user involvement.

**Methods:**

A qualitative study with semi‐structured interviews was conducted. The approach was inductive and open, and data were analysed thematically. Twelve GPs were recruited through snowball sampling and individually interviewed in March–April 2021.

**Results:**

Six themes were generated: Relation and knowledge; Negotiation; Compliance; Information, communication and dialog; Time and process; and GPs' considerations of involvement. The GPs described a plethora of tools and strategies to shape their relationship with the patient, individually inform the patient, and negotiate their power to achieve compliance and, thus, the best treatment for the patient.

**Conclusion:**

GPs consider patient involvement equal to information as a means to compliance to some extent, but they also consider information and compliance interdependent. However, patient involvement is challenging for the GPs when the patient is misinformed or when the GP's current status is poor.

## 1 Background

In recent decades, the role of general practitioners (GPs) has changed significantly [1, 2, 3]. Hospitals are now implementing increased specialization and optimized treatment courses, resulting in shorter admission times and fewer beds [4]. Simultaneously, demographic development towards an increased number of older people and, thus, more people with one or more chronic conditions [5, 6], along with a shortage of healthcare staff, especially in hospitals [7], puts considerable pressure on the healthcare system. In this context, GPs might meet patients when their treatment course is still unfinished [3, 4, 5]. Thus, the GP plays an increasingly important role in the healthcare system, wherein patient involvement is considered a key element in delivering individualized and tailored treatment [5, 6, 8]. For patients, this increased specialization is accompanied by increased complexity in navigating the healthcare system [5], especially in cases of multiple diseases, as they are assigned to different healthcare professionals across specialities, which might result in unstructured, discontinued and unfinished treatment courses [6, 7].

Moreover, patient involvement has become the norm [9], and there is a political expectation that GPs enable patient involvement in the treatment course across sectors, with the unsubtle aim of reducing admissions and readmissions [8]. Thus, the GP's core task is to coordinate treatment courses to ensure they are individualized, effective and coherent [9]. Hence, GPs are often considered patients' gatekeepers to the healthcare system [10].

Furthermore, there is substantial evidence that patient involvement has a positive impact on patient satisfaction and treatment outcomes as accomplished by shared decision‐making and self‐management [11]. However, to our knowledge, GPs' perceptions of user involvement remain unexplored. Hence, this study aimed to explore GPs' perceptions of user involvement and their considered challenges and opportunities regarding user involvement.

## 2 Methods

This study is reported in accordance with the Consolidated Criteria for Reporting Qualitative Research (COREQ), a 32‐item checklist for interviews [12].

### 2.1 Design

To explore GPs' perceptions of user involvement, a qualitative study design with semi‐structured interviews was considered appropriate. The approach was inductive and open, without predefined codes [13].

### 2.2 Setting and Participants

GPs were recruited through purposive sampling. Eligible participants were approached through a gatekeeper comprising the head of research of general practice, who is also a practising GP. From there, snowball sampling was performed, and participants were included with a view to ensuring variation in sex, age, years of experience, practice type (single‐handed/partnership) and geographic location in Denmark. The structure and nature of Danish general practice are described in Box 1. Eligible participants received a written description of the project by email. GPs who showed interest in participating were subsequently contacted by telephone or email to plan the interview.

Box 1 **The structure and nature of Danish general practice**

**Table:** 

Danish health care is tax‐paid and free of charge for the individual patient, based on the principles of free and equal access to healthcare for all citizens. In Demark, there are about 3500 general practitioners (GPs) distributed on about 2000 clinics. Each GP has on average 1600 patients on their list.
With considerable variations across the country, a GP is responsible for between ten and 100 contacts per day of which face‐to‐face contacts count for between ten and 50 contacts per full‐time GP per day. These variations are due to type of practice (solo or multi‐person practice), the doctor's age and the geographical location of the practice, both in terms of remote or urban municipalities and across regions in Denmark. Differences across practices in the number of contacts per full‐time doctor may also be due to differences in the use of practice staff, work organization, and the use of IT technology. However, information about these factors is not immediately available.
The mean age of Danish GPs is 51 years, and 48% identify as women.

No predefined sample size was decided to cover as many nuances as possible, yet with a cautious view to data saturation (meaning that no additional data are found [14] in the completed interviews). A total of 12 individual interviews were carried out. Of the participants, six identified as women and six as men. They were between 43 and 61 years of age (mean: 52 years) and had between 2 and 26 years of experience as a GP (mean: 15 years). They represented both rural and urban locations across Denmark. In the results, they are labeled GP1, GP2 […] GP12. None of the participants were previously known to the interviewer.

### 2.3 Data Collection

Data were collected in March–April 2021. The last author (MM), who identifies as male, has a background as a health professional and is an experienced researcher, conducted the interviews. The interviewer was not previously known to any of the informants.

In‐depth, semi‐structured, individual interviews were conducted. An interview guide was prepared based on a scoping literature review. The main topics of the interview guide were as follows: Introduction/briefing, How do you understand patient involvement (examples), How do you practice involvement (examples), What impact do you consider involvement to have on the patient's course and your relation to the patient, How do you encourage patients to be involved, What challenges and opportunities do you experience related to involvement, How would you suggest involvement to be increased and optimized in general practice, and Debriefing.

The interview guide was pilot‐tested in a single interview and revised accordingly (minor adjustments). The test interview was not included in the analysis. The interviews were conducted by telephone, audio‐recorded and transcribed verbatim for the analysis. The interviews lasted between 13 and 43 min (average: 20 min).

### 2.4 Analysis

A thematic analysis inspired by the work of Thomas [15] was conducted. The method represents a pragmatic approach with the following steps: Step 1 involved coding and the extraction of findings; data extraction was approached inductively, and the extracted data were grouped according to similarity in meaning as judged by all authors. Through this process, descriptive themes emerged (Step 2). In Step 3, analytical themes were generated. Relationships between the descriptive and defined analytical themes were evaluated, gathering the descriptive themes into fewer major groups (themes). The final themes reflect a consensus among all authors.

In reporting the findings, the descriptive themes are primarily presented, including the number of codes and analytical themes constructing the respective themes. However, when the data include illustrative quotations to support the themes, these quotations are also presented.

### 2.5 Ethics

The study complied with the ethical principles for medical research as described in the Declaration of Helsinki [16] and the General Data Protection Regulation (GDPR) legislation [17]. The study was approved by the Research Ethics Committee of the University of Southern Denmark (ID 21/8757) and the Committee of Multipractice Studies in General Practice (part of the Danish Society for General Practice; ID MPU 10‐2021). The participants were informed of the purpose of the study both verbally and in writing, and verbal consent was obtained. The participants were also informed that the interviews would be audio‐recorded and that all identifying information would be anonymised in the transcripts. The project was financially approved by Danish Regions. No further approval was needed.

## 3 Results

**Figure 1:**
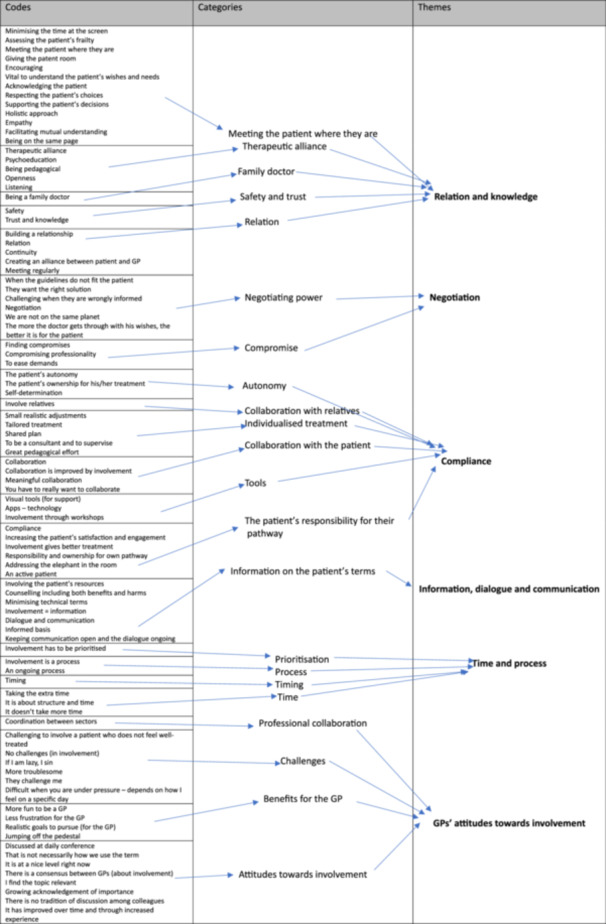
Code tree.

During Step 3 of the analysis process, six descriptive themes were generated: *Relation and knowledge; Negotiation; Compliance; Information, communication and dialog; Time and process;* and *GPs' considerations of involvement*. The analytical themes and the codes behind these descriptive themes are shown in Figure 1.

### 3.1 Relation and Knowledge

The informants evolved continuously regarding their relationship with their patients, and the theme of *Relation and knowledge* represents a variety of codes and categories. The GPs considered it crucial for successful involvement to be aware of and understand the patient's here‐and‐now situation regarding, for example, their frailty and their wishes and needs. The GPs would use this information to meet the patient where they were with a holistic and empathetic approach, meaning that they wanted to signal respect for the patient's choices and decisions:

An empathetic and respectful relationship between the doctor and the patient is essential for good collaboration on the treatment.

(GP7)

Being eager to acknowledge and support the patient's decisions, the GPs also expressed a high level of awareness about their own behavior in the meeting, including, e.g., minimizing time at the screen and giving the patients room to express their wishes and needs:

I think that it is easy to fill in database information instead of starting by hearing what the patients say and where they are in their lives.

(GP10)

and

This involves giving the patients information and requires that I, as a doctor, give them space to have their say in relation to the treatment.

(GP8)

Thus, the GPs considered it vital to be on the same page as the patients for involvement to be successful. This was also described by the informants as a therapeutic alliance—some used terms such as “psychoeducation” or “being pedagogical”, while others described how vital they considered it to be open and listen to the patients:

It involves listening to the patient's needs, preferences and concerns…

(GP2)

Basically, a relationship built on trust and knowledge, continuity, and an alliance with the patient—according to the GPs, the essence of being a family doctor—was considered the core of involvement.

### 3.2 Negotiation

The theme of *Negotiation* was closely related to the former theme, *Relation and knowledge*. According to the GPs, there is an ongoing negotiation aimed at caring about the relationship and making sure that the GP and the patient are on the same page regarding knowledge. Negotiation could, for example, be necessary when the GP and the patient are “*…not on the same planet…”* or when the patient, according to the GP, is *“wrongly informed”*. One of the categories behind the theme is *Negotiating power*—it does, however, remain unclear whether this concerns a real negotiation of power between the patient and the GP or is an inner consideration for the GP to decide how much power they are ready to give up achieving a certain goal:

Sometimes, after all, it is…kind of [a] negotiation regarding what might be professionally best and what the patient wants to participate in.

(GP3)

and

The more the doctor gets through with his wishes, the better it is for the patient […] …so it [involvement] is quite obviously beneficial for the patients.

(GP9)

However, regardless of whether the negotiation was real or internal, the GPs were concerned with patients getting the best possible treatment, and in cases where they found the patients to be wrongly informed or the guidelines did not fit a specific patient, they were ready to ease demands and find a compromise, even though they might feel they were compromising their professionalism:

I probably also sometimes compromise with my professionalism…

(GP9)

Thus, for the GPs, patient involvement could imply reduced power or “getting off the pedestal”. It was considered a means to an end—that is, the best treatment for the patient while accounting for the relationship.

### 3.3 Compliance

Interwoven with the former themes, the theme of *Compliance* was constructed. During the interviews, compliance was ubiquitous—both mentioned as a specific term and more indirectly as the overall goal for the treatment to be successful. According to the GPs, compliance and involvement are intricately connected and somehow interdependent—without compliance, there is no involvement and vice versa. Compliance appeared to be an obvious marker of the GPs' work and relationship with the patient but was also a complex phenomenon for the GPs to facilitate. Hence, the GPs mentioned the importance of acknowledging the patient's autonomy and self‐determination to strengthen the patient's ownership of their treatment:

The purpose of patient involvement is to strengthen patients' self‐determination, improve their treatment (via involvement) and increase their satisfaction.

(GP7)

and

Well, it [involvement] is increased knowledge, insight and ownership over one's illness and suffering.

(GP6)

To facilitate this ownership (i.e., compliance), the GPs used a variety of tools, such as collaboration with relatives, tailored treatment and a shared plan.

It is also about involving relatives if the patient wishes so.

(GP5)

Moreover, the GPs mentioned the need for great pedagogical effort and serving as a consultant and supervisor—all to facilitate and cherish meaningful collaboration with the patient:

This [involvement] can be done by listening to their wishes and needs and trying to build a collaboration that is meaningful, also for the patient.

(GP8)

They did, however, also mention that this collaboration could be challenged:

As a GP, it is also important to have clear communication—and then you really have to want to cooperate.

(GP5)

and

Addressing the elephant in the room…

(GP1)

Thus, according to the GPs, collaboration was paramount and the very basis for involvement. On the other hand, they also considered involvement necessary for collaboration.

### 3.4 Information, Communication and Dialog

In the code tree (Figure 1), the theme of *Information, communication and dialog* appears to be narrow, with relatively few codes and only a single category. Information, communication and dialog were, however, mentioned repeatedly by all informants across all interviews, and their consistency in describing them was striking. Overall, the GPs had two takes on information, communication and dialog. First, they should be on the patient's terms:

Patient involvement, in my opinion, means that you as patient and doctor meet on as equal a level as possible.

(GP6)

and

Well, […] I meet them as they meet me…

(GP9)

Hence, numerous quotations focused on the GPs' efforts to meet the patients on their terms or level, meaning that the GPs had to adjust and adapt their information, communication and dialog according to each patient to ensure that the patient was adequately informed. This leads to the second take on this theme as the GPs considered information, communication and dialog to be involved:

… good information, actually. […] and information about their chronic diseases and what is going to happen now and in the longer term.

Most of all, a dialog about their own responsibility…

(GP1)

This notion of involvement explains the GPs' overall focus on individually tailored information and an equitable dialog.

### 3.5 Time and Process

The theme of *Time and process*, like the previous theme, had many layers per the GPs' understanding. During their description of involvement, they revolved around time and process in the sense of *“taking the necessary time”* to explain something, create a trusting relationship, or similar. They talked about timing, meaning that it is important not to force anything but to wait for the correct time for the patient to be ready for lifestyle changes despite their professional knowledge telling them that an immediate change would be preferable:

I have found peace with myself… […] …that this is how it is. I cannot get through to the patient by now with more [changes].

(GP11)

Thus, in the GPs' understanding, this theme was intricately connected to compliance as time and process are necessary to provide a place for understanding to increase compliance.

However, the informants were also specifically asked whether they found that involvement was time‐consuming. As shown in Figure 1, their answers varied substantially; some found that involvement did not take more time compared to not involving the patient, whereas others found involvement rather time‐consuming. These varying answers might reflect the fact that, according to the GPs, involvement is a matter of compliance achieved through individualized and tailored information and a trusting relationship built over time. This means that involvement inevitably will take more time than non‐involvement, both in the here and now in terms of time required to inform and explain and in terms of the ongoing process “over time”.

### 3.6 GPs' Attitudes Towards Involvement

The final theme, the GPs' attitudes towards involvement, was not so much a theme as a meta‐perspective on the data combined with the informants' reflections when asked whether they discuss involvement with their colleagues. Some informants described involvement as challenging and troublesome and indicated that it was an extra task they had to perform:

This is also the case, one might say, it depends on my current shape. So, what I think, if you have to practice patient involvement on a larger scale, you have to create a mindset. Create a method that, regardless of how tired, unmotivated or stressed you are, whether you have been arguing with the wife or not, you use a tool to get around it, to get the patient involved in what we are doing. Because, if the patient does not join the process, the success rate will decrease.

(GP6)

On the other hand, they also associated involvement with less frustration for the GP, more realistic goals to pursue and more fun for the GP:

Then I find that what we end up with is realistic. I think i.e. probably the biggest thing that I experience, that my patients come back again and dare to come back… […] …it makes being a doctor a little more fun.

(GP10)

Regarding the discussion of involvement with their colleagues, overall, the informants agreed that it was not necessary as *“It is at a nice level, right now”*. A single informant stated that involvement was discussed at a daily conference, but the majority of the GPs did not, despite an increasing acknowledgement of the importance of involvement:

Well…I guess there isn't a tradition for that. In other words, I am sure that we, as general practitioners, involve the patients much more… […]. But I do not discuss it regularly or routinely with my colleague.

(GP8)

Moreover, none of the informants had ideas for improving or increasing involvement in general practice:

I think it's at a nice level now. At least here with us, or should I then hand over the responsibility for the treatment to the patients? And they shouldn't have this responsibility, it's my responsibility. And…they are fully involved in the treatment now, so I think it's at a good level.

(GP1)

### 3.7 Discussion

In this study, we explored GPs' perceptions of patient involvement and generated six descriptive themes. The themes were interwoven and revolved around a genuine wish to do the best for the patients, including *Relation and knowledge; Negotiation; Information, communication and dialog;* and *Time and process* as a means to reach the overall goal of *Compliance*. Thus, for some informants, patient involvement equalled information, whereas others considered information and compliance to be two sides of the same coin—no involvement without compliance and vice versa. Considering an “equal to” sign between information and involvement is quite common among health professionals, as reported in a plethora of studies in the field [1, 5, 6, 8]. Despite involvement being more than information, information is considered a crucial prerequisite for patients to actively participate in their treatment and care [18, 19]. According to Coulter et al. (2006), patient‐centeredness and involvement include acknowledging patients as active partners in their pathways, and with GPs' continuous focus on compliance, including codes such as autonomy, ownership and self‐determination, the GPs in this study revealed a nuanced and rich understanding of patient involvement.

However, room for improvement remains. Despite the aforementioned nuanced notions of patient involvement, the GPs still expressed an “add‐on consideration” of involvement as their performance regarding involvement depends on how they feel—their current status. This means that GPs can choose not to involve the patients if their current status is poor or add involvement as they please, which has also been reported previously [19]. Moreover, the fact that the informants considered the level of involvement in general practice “nice” and found no reason to improve it could be somewhat disturbing, reflecting a professional culture embedded in a biomedical model [20] or a reluctance rooted in a lack of confidence and knowledge about involvement, also reported in other studies regarding GPs' willingness to tailor their care to individual needs [21]. The latter is supported by our findings indicating that there was no tradition among the GPs to discuss patient involvement with their colleagues. One informant (GP12) stated that they would occasionally discuss involvement with the nurses in the clinic but never with their GP colleagues (data not shown).

Despite the GPs' consideration of patient involvement as necessary for compliance, they were challenged by the balance between the individual patient's information level and wishes and their own professionalism and knowledge. This dilemma has been found to have several consequences for the physician‐patient relationship, including time spent to re‐inform, feelings of distrust from patients and challenged authority [22]. This could also point to a more deeply felt ethical challenge for the GPs as they as medical doctors have sworn to the Hippocratic oath of doing no harm [23]. This means that the negotiation between the patient's wishes and the GP's professional knowledge compromises the GP's “act of profession” as described by Pellegrino (1981). Accordingly, the patient‐relationship should be characterized by competence, informing the patient and getting consent [16]. Despite being a bit outdated, Pellegrino might point to a core dilemma for the GPs and a deeply felt ethical challenge for them and thus, not simply a matter of power negotiation or lack of tradition to discuss and consider user involvement.

### 3.8 Limitations and Strengths

We acknowledge some limitations of this study. The snowball sampling approach was a pragmatic strategy and might have compromised socio‐demographic variation as we welcomed each possible informant without strategic considerations. On the other hand, our sample varied greatly regarding sex, age, experience as a GP, and locations across Denmark (rural and urban). Furthermore, none of the participants were previously known to the interviewer; thus, there was no power imbalance or risk to the informants, ensuring that the participants felt sufficiently safe and able to openly provide data that were honest and valuable in their candor.

The number of participants was neither pre‐defined nor optimal but reflected a pragmatic approach to sample size. However, given the results, we do not consider this a severe limitation as we generated sufficient data and found both nuances and patterns to appropriately address our research question. Moreover, our team includes a patient representative (NAS) who participated actively in data analysis (transcription and formal analysis) and reviewed the results section for recognizability and societal relevance, which we consider a considerable strength of our study.

Nevertheless, it remains unclear whether further interviews would have contributed unexpected results and new knowledge.

Regarding validity and whether the study explored and reflected on its research question, conducting a pilot test of the interview guide was helpful because the quality of data generated through interviews depends largely on the wording of the questions and the interviewer's skills and experience. In the present study, all interviews were conducted by the last author, who is an experienced interviewer; moreover, the interview guide was pilot‐tested, which we consider a strength of the study.

## 4 Conclusion

This interview‐based study revealed that GPs consider patient involvement equal to information as a means to obtain compliance and that they consider information and compliance interdependent. The GPs described a plethora of tools and strategies to shape their relationship with the patient, individually inform the patient, and negotiate their power to achieve compliance and, thus, the best treatment for the patient. However, patient involvement is also challenging for GPs when the patient is misinformed or when the GP's current status is poor.

Our study contributes with vital information regarding the complexity of GPs perceptions of user involvement and their continuous effort to understand and implement user involvement in their practise. We do, however, recommend that further research with a larger sample size be conducted to confirm our findings on this topic.

## Declaration of Generative AI

Declaration of the use of generative AI and AI‐assisted technologies in the writing process.

## Author Contributions

Conceptualization: Michael Marcussen and Birgitte Nørgaard. Data curation: Birgitte Nørgaard, Nanna Aarup Skotte, Elisa Simonsen, and Michael Marcussen. Formal analysis: Michael Marcussen and Birgitte Nørgaard. Funding acquisition: Michael Marcussen and Birgitte Nørgaard. Investigation: Michael Marcussen and Birgitte Nørgaard. Methodology: Michael Marcussen and Birgitte Nørgaard. Project administration: Michael Marcussen and Birgitte Nørgaard. Resources: Michael Marcussen and Birgitte Nørgaard. Software: Michael Marcussen and Birgitte Nørgaard. Supervision; Birgitte Nørgaard and Michael Marcussen. Validation: Birgitte Nørgaard, Nanna Aarup Skotte, Elisa Simonsen, and Michael Marcussen. Visualization: Birgitte Nørgaard, Nanna Aarup Skotte, Elisa Simonsen, and Michael Marcussen. Roles/Writing – original draft: Birgitte Nørgaard. Writing – review and editing: Birgitte Nørgaard, Nanna Aarup Skotte, Elisa Simonsen, and Michael Marcussen. All authors contributed substantially to this paper.

## Disclosure

The authors have nothing to report.

## Conflicts of Interest

The authors declare no conflicts of interest.

## Data Availability

Data is available by resonable request to the corresponding (first) author.

## References

[1] M. Marcussen , S. B. Titlestad , K. Lee , N. Bentzen , J. Søndergaard , and B. Nørgaard , “General Practitioners' Perceptions of Treatment of Chronically Ill Patients Managed in General Practice: An Interview Study,” European Journal of General Practice 27, no. 1 (2021): 103–110. 34078226 10.1080/13814788.2021.1932810 PMC8183538

[2] K. Lee , S. B. Titlestad , B. Nørgaard , N. Bentzen , J. Søndergaard , and M. Marcussen , “Patient Perspectives on the Management of COPD and Type 2 Diabetes in General Practice: An Interview Study,” BMC Primary Care 23, no. 1 (2022): 174. 35836109 10.1186/s12875-022-01787-8 PMC9284705

[3] L. B. Larsen , A. L. Sonderlund , J. Sondergaard , et al., “Targeted Prevention in Primary Care Aimed at Lifestyle‐Related Diseases: A Study Protocol for a Non‐Randomised Pilot Study,” BMC Family Practice 19, no. 1 (2018): 124. 30031380 10.1186/s12875-018-0820-8 PMC6054846

[4] Z. A. Abdalkareem , A. Amir , M. A. Al‐Betar , P. Ekhan , and A. I. Hammouri , “Healthcare Scheduling in Optimization Context: A Review,” Health and Technology 11, no. 3 (2021): 445–469. 33868893 10.1007/s12553-021-00547-5 PMC8035616

[5] B. Nørgaard , S. B. Titlestad , and M. Marcussen , “Patients' Perspectives on Involvement in General Practice: A Thematic Analysis of Free‐Text Comments,” Journal of Evaluation in Clinical Practice 29 (2023): 816–821. 37143436 10.1111/jep.13858

[6] S. B. Titlestad , M. Marcussen , M. S. Rasmussen , and B. Nørgaard , “Patient Involvement in the Encounter Between General Practice and Patients With a Chronic Disease. Results of a Scoping Review Focusing on Type 2 Diabetes and Obstructive Pulmonary Disease,” European Journal of General Practice 28, no. 1 (2022): 260–269. 36503359 10.1080/13814788.2022.2153827 PMC9754033

[7] G. L. Anesi and M. P. Kerlin , “The Impact of Resource Limitations on Care Delivery and Outcomes: Routine Variation, the Coronavirus Disease 2019 Pandemic, and Persistent Shortage,” Current Opinion in Critical Care 27, no. 5 (2021): 513–519. 34267075 10.1097/MCC.0000000000000859 PMC8416747

[8] B. Nørgaard , S. B. Titlestad , and M. Marcussen , “Shared Decision‐Making in General Practice From a Patient Perspective. A Cross‐Sectional Survey,” Scandinavian Journal of Primary Health Care 40, no. 2 (2022): 167–172. 35481437 10.1080/02813432.2022.2069700 PMC9397466

[9] J. Russell , N. Fudge , and T. Greenhalgh , “The Impact of Public Involvement in Health Research: What Are We Measuring? Why Are We Measuring It? Should We Stop Measuring It?,” Research Involvement and Engagement 6 (2020): 63. 33133636 10.1186/s40900-020-00239-w PMC7592364

[10] A. R. Machin , “Gps Are Far More Than Gatekeepers,” British Journal of General Practice 73, no. 729 (2023): 167. 10.3399/bjgp23X732381 PMC10049601 36997208

[11] A. Coulter , “Patient Engagement‐‐What Works?,” Journal of Ambulatory Care Management 35, no. 2 (2012): 80–89. 22415281 10.1097/JAC.0b013e318249e0fd

[12] A. Tong , P. Sainsbury , and J. Craig , “Consolidated Criteria for Reporting Qualitative Research (COREQ): A 32‐item Checklist for Interviews and Focus Groups,” International Journal for Quality in Health Care 19, no. 6 (2007): 349–357. 17872937 10.1093/intqhc/mzm042

[13] A. Bingham and P. Witkowsky , “Qualitative Analysis: Deductive and Inductive Approaches,” in Analyzing and Interpreting Qualitative Data: After the Interview, ed. C. Vanover , P. Mihas , and J. Saldaña (SAGE Publications, 2022), 133–146.

[14] G. Guest , E. Namey , and M. Chen , “A Simple Method to Assess and Report Thematic Saturation in Qualitative Research,” PLoS One 15, no. 5 (2020): e0232076. 32369511 10.1371/journal.pone.0232076 PMC7200005

[15] J. Thomas and A. Harden , “Methods for the Thematic Synthesis of Qualitative Research in Systematic Reviews,” BMC Medical Research Methodology 8 (2008): 45. 18616818 10.1186/1471-2288-8-45 PMC2478656

[16] E. D. Pellegrino , “A Philosophical Basis of Medical Practice: Toward a Philosophy and Ethic of the Healing Professions,” Oxford University Press, College Edition (January 8, 1981).

[17] The General Data Protection Regulations (GDPR) , Regulation (EU) 2016/679 of the European Parliament and of the Council of 27 April 2016 on the Protection of Natural Persons With Regard to the Processing of Personal Data and on the Free Movement of Such Data, and Repealing Directive 95/46/EC (General Data Protection Regulation) OJ L 119/1, (2016).

[18] A. Coulter and J. Ellins , “Patient‐Focused Interventions: A Review of the Evidence: Health Foundation London,” (2006).

[19] C. Weber and B. Nørgaard , “Nurses' Perspectives on Patient Involvement in an Emergency Department–An Interview Study,” International Emergency Nursing 72 (2024): 101401. 38198947 10.1016/j.ienj.2023.101401

[20] A. Negri , C. Zamin , G. Parisi , A. Paladino , and G. Andreoli , “Analysis of General Practitioners' Attitudes and Beliefs About Psychological Intervention and the Medicine‐Psychology Relationship in Primary Care: Toward a New Comprehensive Approach to Primary Health Care,” Healthcare 9, no. 5 (May 19, 2021): 613, 10.3390/healthcare9050613. 34069738 PMC8161354

[21] S. M. Vanderhout , M. Bhalla , A. Van , et al., “The Impact of Patient and Family Engagement in Child Health Research: A Scoping Review,” Journal of Pediatrics 253 (2023): 115–128. 36179891 10.1016/j.jpeds.2022.09.030

[22] Q. Lu and P. J. Schulz , “Physician Perspectives on Internet‐Informed Patients: Systematic Review,” Journal of Medical Internet Research 26 (2024): e47620. 38842920 10.2196/47620 PMC11190621

[23] B. Varkey , “Principles of Clinical Ethics and Their Application to Practice,” Medical Principles and Practice 30, no. 1 (2021): 17–28. 32498071 10.1159/000509119 PMC7923912

